# Temporal and spatial characterization of physiological noise in rs-fMRI at a high temporal resolution

**DOI:** 10.1038/s41598-025-31018-w

**Published:** 2025-12-06

**Authors:** Olga Kuldavletova, Marin Mauboussin, Mikael Naveau, Anais Vandevelde, Nicolas Delcroix, Marc Joliot, Olivier Etard

**Affiliations:** 1 Normandie Université, UNICAEN, INSERM, COMETE, CYCERON, CHU Caen, Caen, France; 2 Normandie Université, UNICAEN, CNRS, INSERM, CYCERON, Caen, UAR3408/US50 France; 3 CHRU de Tours, Tours, France; 4 GIN, IMN, UMR5293, CEA, CNRS, Université de Bordeaux, Bordeaux, France; 5 Universite de Caen Normandie, 2 rue des Rochambelles, Caen, 14000 France

**Keywords:** FMRI noise, Physiological noise, Respiratory cycle, Cardiac cycle. cerebral blood flow, Neuroscience, Cardiovascular biology, Respiration

## Abstract

The fMRI signal contains “noise” components resulting from physiological processes that interfere with the component due to neuronal activation and obscure the understanding of brain function. Having the neuronal activation component as the unknown, it is crucial to characterize the spatial and temporal aspects of the signal component that is due to cardiac and breathing cycles. 17 fMRI exams were performed in 9 subjects with a 3T Philipps MRI scanner with high sampling frequency (repetition time 125 ms, one slice). Photoplethysmography was used to track the cardiac cycle, and a pneumatic thoracic respiration transducer was used to measure the breathing cycle. The impact of the physiological signal on fMRI signal was evaluated in four regions of interest: (1) a region encompassing all areas below the subarachnoid space (Global Signal - GS), (2) ventricles (CSF), (3) venous return (VR), and (4) the region surrounding Middle Cerebral Arteries (MCA). Physiological noise is found heterogeneous across the ROIs in terms of amplitude and phase. The impact of respiration on the fMRI signal is greater than that of the heart, and it may be mediated by modulation of the venous return.

## Introduction

fMRI is a useful imaging tool that allows visualization of neuronal activity based on Blood Oxygenation Level Dependent (BOLD) effect. However, alongside the neuronal component of interest, fMRI signal contains “noise” components from movement or physiological processes such as heartbeat and breathing. These components can interfere with the component of interest due to neuronal activation and obscure the conclusions regarding neuronal activation of the brain. Having the neuronal component of the signal as the unknown, we need to know what the noise component looks like to be able to judge if the signal is properly de-noised. There is currently no consensus on the optimal method for removing physiological noise from fMRI data. The effectiveness of these methods critically depends on the nature of the noise, whether it is high or low frequency, and its spatial homogeneity. In a previous study we have shown that using different methods for de-noising the fMRI data can completely modify or even invert the conclusions about the neuronal activation in which studies are interested^1^. Therefore, it is necessary to characterize spatially and temporally the component in the signal, which is due to physiological processes such as cardiac and breathing cycles.

There are usually two types of the physiological noise considered in the imaging studies. Some studies modeling the physiological noise in fMRI data using the Physiological Response Functions (PRF) show that cardiac and breathing activities result in low-frequency noise of around 0.1 Hz in fMRI data in gray matter regions. This noise originates not from the respiratory and cardiac cycles by themselves, but from the homeostatic physiological variations, like heart rate variability and respiratory flow variations^2–4^. Other studies addressing physiological noise concentrate on the cardiac and breathing artifacts in the high frequencies of around 0.3–1 Hz, with high-frequency cardiac artifacts due to cardiac pulsations around 1 Hz^5^, and breathing artifacts around 0.3–0.4 Hz^6^. This high-frequency noise is addressed by RETROICOR and related methods^7^, filtering out signals that are synchronized to cardiac pulses and breathing cycles.

Before modeling any signal, we need to know what it looks like, so it can be practical first to visualize and characterize it as directly as possible. It is possible to do this using an averaging technique that allows for revealing the events synchronized to a trigger or a cycle, while averaging the non-synchronized noise to zero. This method is convenient to all time series where a specific event or cycle needs to be characterized and is widely used in physiology and in EEG, however, its use is relatively rare in imaging. This method can characterize the high-frequency physiological nuisances due to breathing cycle and cardiac pulsations.

The characterization of the physiological noise component might also allow us to uncover the underlying physiological processes that lead every breath and heartbeat to induce variations in the fMRI signal. Previous studies attempted to directly measure and characterize physiological effects on fMRI signal, and they are mainly focused on the cardiac-related component. A study by Dagli and collaborators^5^ has characterized spatial distribution of cardiac-related noise in the brain in the fMRI signal. For this purpose, they retrospectively reconstructed the cycle, by sorting images according to the phase of the cardiac cycle at their acquisition time to acquire the signal intensity of voxels during a full cardiac cycle. Their study focused only on cardiac-related noise and presented only the spatial distribution of the areas most affected by the cardiac noise, but not the temporal and phase characterization of the cardiac-related signal. A recent study^8^ has characterized the coupling between the cerebrovascular oscillations and the flow of cerebrospinal fluid (CSF). The article described the physiological processes linking the flows of blood and the CSF in the brain, however without linking it to the cardiac or respiratory activity. Another recent study^9^ also focused on the characterization of cardiac-related fMRI fluctuations. This article presents both spatial distribution of the cardiac noise, and the temporal course of the cardiac-related fMRI oscillations. Due to the presence of aliasing, these studies, however, retrospectively reconstruct the cycle from multiple acquisitions that fell at different phases of the cardiac cycle, in order to obtain the cardiac-related oscillation of the fMRI signal. This reconstruction was necessary due to the technical difficulty of directly measuring the cardiac effect on the fMRI signal oscillation. Such measurements require a high acquisition rate (above 2 Hz), which is inaccessible for most fMRI studies that scan the whole brain (classically, around 1 Hz). An MRI technique called magnetic resonance encephalography (MREG) can achieve high-speed functional imaging and measure fluid dynamics^10,11^, however, the main goal of our study is to characterize the cardiac and respiratory impact on the fMRI signal that can be similar to classic fMRI studies evaluating resting state or cognition-related activity. One potential solution to circumvent the technical limitations is to reduce the TR by selecting a single slice for study instead of the entire brain. This can be unsuitable for studying cognitive function; however, this can be a way to directly measure the effect of a cardiac cycle on the fMRI.

The studies that tried to extract the respiratory effect from the fMRI signal focused on the estimations of respiratory flow or flow variations. They found the fluctuations in the fMRI signal linked to the fluctuations in respiratory flow^2,4,12^.

The characterization of the fMRI response to the systole and diastole and to inspiration and expiration would be useful for understanding through what mechanisms physiological processes affect fMRI signal and might allow us to adjust noise-reduction techniques. To attempt this characterization, we employed ultrashort TR (125 ms) to measure the fMRI signal in distinct brain regions and subsequently averaged it using cardiac and respiratory cycles as triggers. We postulated that the portion of the MRI signal synchronized with the cardiac and respiratory cycles would be revealed by this back-averaging procedure, allowing us to characterize the amplitude and phase of these oscillations in distinct brain regions.

## Materials and methods

### Participants

9 participants (age 32.3 ± 11.1 years old, 3 female) with no history of neurological diseases were recruited for this study. Informed consent was obtained from all the participants before participation. This study was approved by the National Ethics Committee *(Comité de Protection des Personnes du Sud-Ouest et Outre-Mer 4 ID-RCB #2018-A03281-54*) and was performed in accordance with the Declaration of Helsinki.

### Acquisition

17 MRI exams were performed (1 to 3 sessions per subject, conducted on different days), with a 3 T Philipps MRI scanner including an anatomical 3D T1 sequence (except for one subject) and a 2D single slice EPI-GE sequence (TR = 125 ms, TE = 30 ms, flip angle = 22°, 5 min 12 s per session). Single slice method was chosen to be able to observe the fluctuations in the fMRI signal with the TR low enough to detect cardiac cycle without aliasing. The position of the single-slice acquisition was individually defined according to the following criteria: the slice had to be perpendicular to the sagittal plane and pass through two anatomical landmarks defined on the sagittal slice: (i) the most anterior point of the corpus callosum and (ii) the midpoint between the anterior end of the tentorium cerebelli and the superior end of the cerebellum. These landmarks were chosen because the slice thus defined contained regions composed of the tissues of interest such as the third and lateral ventricles, the vein of Galen, the straight sinus and the mid cerebral arteries. Since head-movement correction algorithms are not suitable for single-slice acquisitions, particular care was taken to limit head movements during acquisition by padding the head with foam in the head coil, instructing participants, and minimizing any discomfort during installation. Before processing, all slices were individually checked for movement before being included in the data to be analyzed. MRI-compatible photoplethysmographie (PPG) sensor placed at the pad of the middle finger of the left hand was used to track the cardiac cycle, and a pneumatic thoracic respiration transducer placed around the abdomen was used to measure the breathing cycle (Biopac Acknowledge). Both signals were synchronized with the MRI acquisition using scanner trigger.

### Analysis

**Fig. 1:**
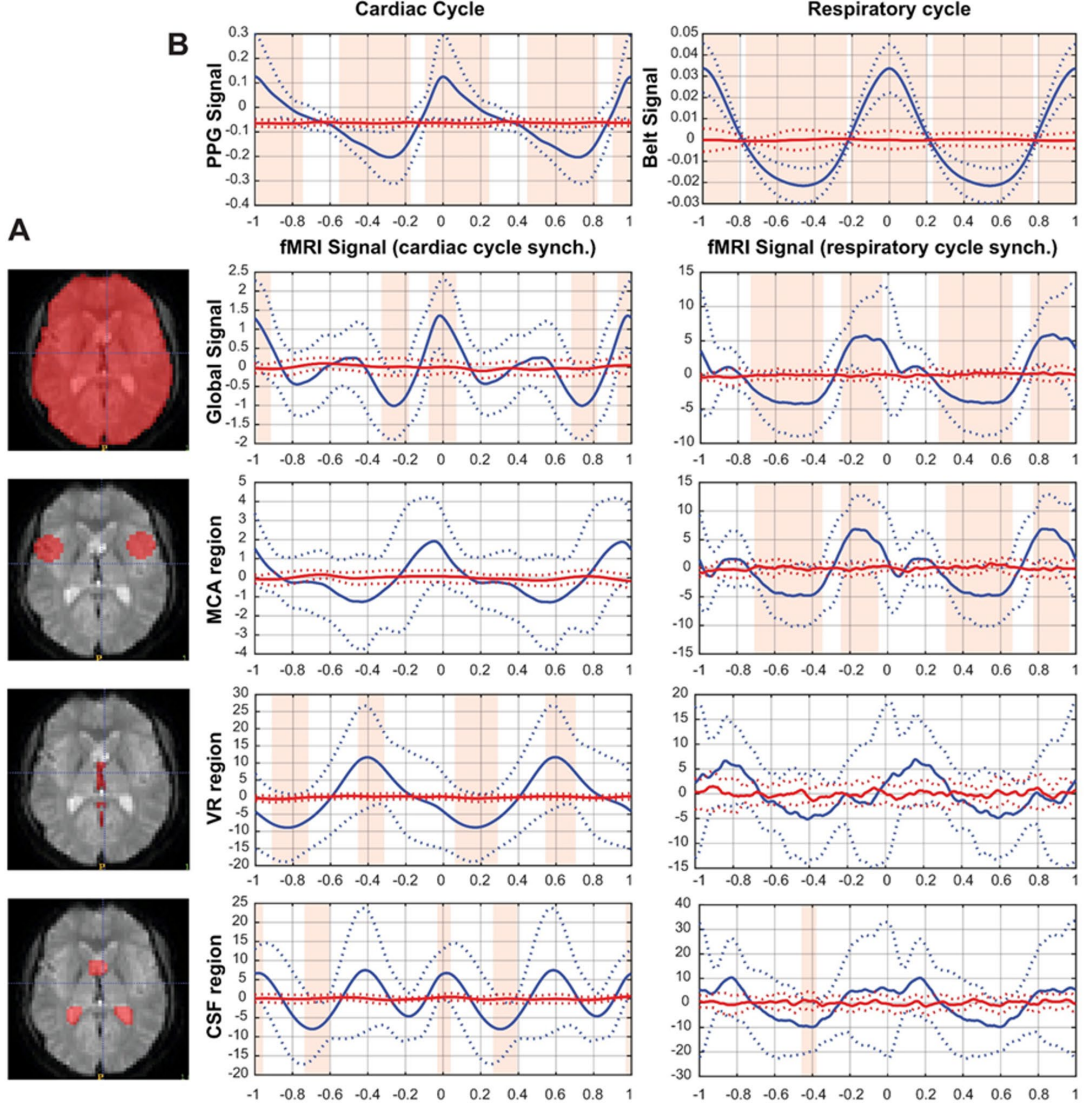
(**A**): Example of the 4 individually determined ROI (in one subject): (1) Global Signal; (2) MCA – area around the middle cerebral artery; (3) VR - venous return region (4) CSF – cerebrospinal fluid region located in the ventricles, regions delineated manually for every subject. (**B**): The physiological and fMRI cycles averaged across subjects. In blue - synchronized grand means (two cycles shown), normalized by subtracting the mean value, of the cardiac, respiratory and fMRI signals at each ROI (average in solid line, SD in dotted line); in red - random grand means, normalized by subtracting the mean value, of the cardiac, respiratory and fMRI signal at each ROI. Shaded areas indicate the intervals in which the synchronized and random grand means are significantly different.

The impact of physiological signals was assessed in four ROIs that were drawn individually using the anatomical T1 slice corresponding to the T2* acquisition (see Fig. 1A): GS region corresponds to all areas below the subarachnoid space; CSF region corresponds to the lateral ventricles and, when present in the single-slice acquisition of some subjects, the third ventricle; VR region corresponds to the venous return provided in this single-slice acquisition by the vein of Galen and the straight sinus; MCA region corresponds to a circle centered on the branches of the middle cerebral artery left and right. The fMRI time series of each voxel were then averaged for each ROI. We used two approaches to analyze the data: in time and frequency domains. The analysis in time domain is used to find the time course and phase relations of the average effect of physiological cycles on the fMRI signal in each ROI. The analysis in frequency domain is used (1) to find the spatial distribution of the cardiac and respiratory effects and (2) to compare the importance of the respiratory and cardiac cycles in each ROI.

In the time domain, all respiratory and cardiac cycles for each subject were automatically detected on their 1 kHz-sampled physiological signals. We used the pulse peak for the cardiac signal and the inspiratory peak for the respiratory signal as triggers. All detected events were then individually checked and manually corrected when necessary, using custom software, and the fMRI signal was resampled at the same sampling rate as the physiological signal (1 kHz) without temporal filtering. To account for heart rate variability, we normalized the time axis of the physiological and fMRI signals so that all heart cycles had the same relative duration with the help of the nearest neighbor interpolation to resample the evenly spaced cycle. We calculated the within-subject mean of the fMRI signal of each ROI aligned by the peak of each cardiac cycle (approximately 421 ± 94 cycles averaged per subject). As all cycles are aligned to the pulse peak, we refer to this as the synchronized mean. A control mean was calculated from the same data to obtain the mean of the fMRI time series not synchronized with the cardiac cycles. This was done in the same way as the synchronized mean, but the start of each cycle was shifted by a random interval within one cycle interval, we will refer to it as the random mean. Finally, the synchronized and random means of the physiological and fMRI signals obtained from each subject (*N* = 17) were averaged into a grand mean (Fig. 1B). These synchronized and random grand means were compared point by point with a multiple t-test corrected for false discovery rate (Fig. 1B, shaded areas). The same procedure was performed for the respiratory cycles (approximately 84 ± 22 cycles averaged per subject).

**Fig. 2:**
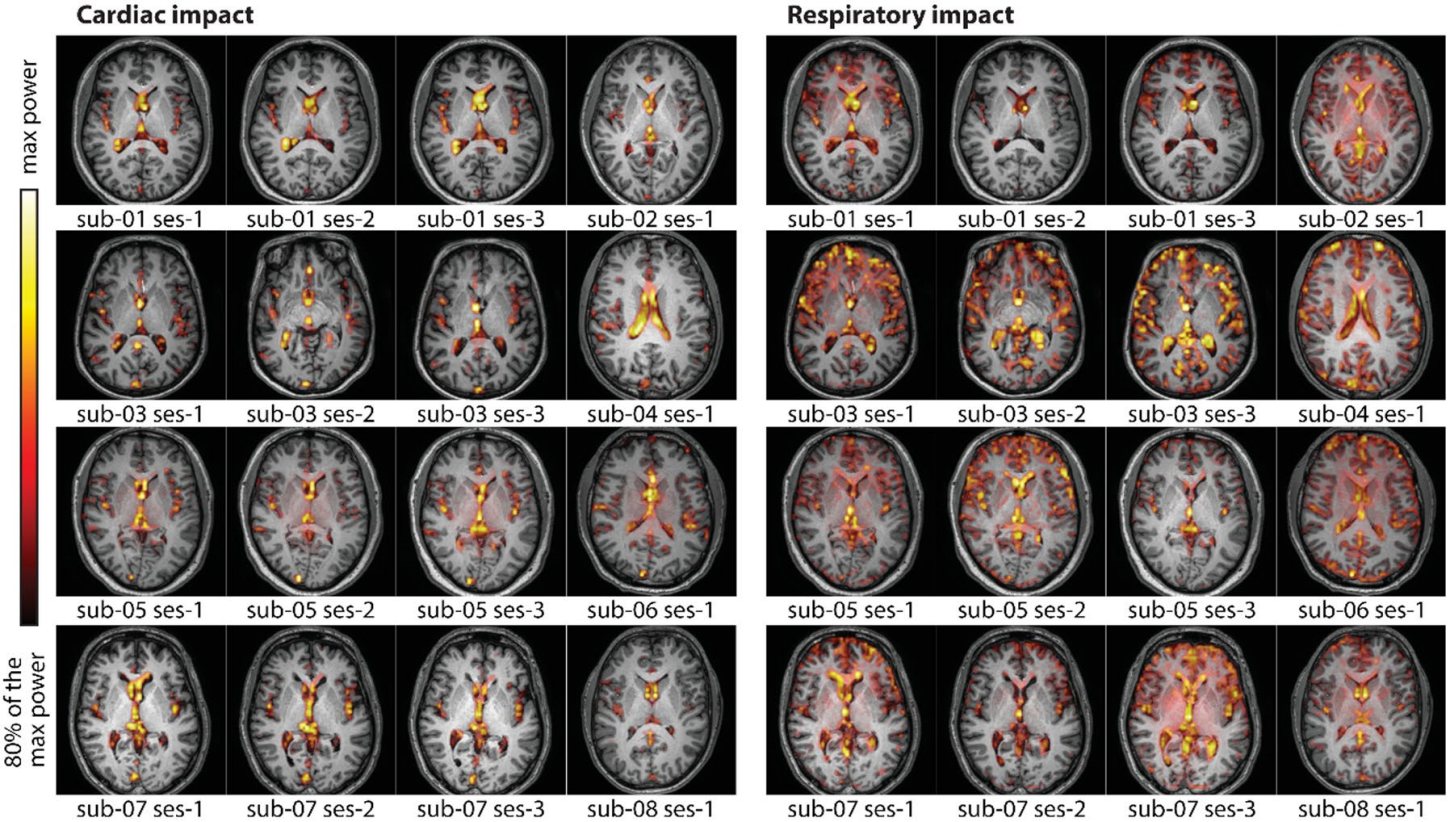
The power of the fMRI signal at the cardiac and respiratory frequencies is overlaid on top of the T1w anatomical image of each participant (except one for whom the T1w image is lacking). The power is log-transformed and normalized subjectwise by the max power. Only the values above 80% of the max power are shown.

**Fig. 3:**
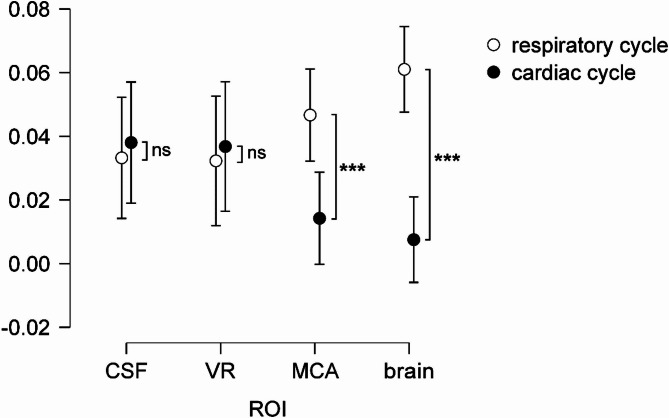
Normalized power of the fMRI signal at cardiac cycle frequency and respiratory cycle frequency in each ROI (*** *p* ≤ 0.001). The bars are the 95% CI. CSF – ventricles; VR – venous return; MCA – the area around the Middle Carotid Artery; GS – Global Signal or whole brain region.

Frequency analysis was performed on a voxel-by-voxel basis to more precisely examine the regions affected by physiological noise (Fig. 2). However, as a voxel-by-voxel comparison between subjects is not possible with this single-slice approach, we also performed a ROI-wise approach to test the difference in amplitude of physiological noise in the respiratory and cardiac frequency bands (Fig. 3). First, the average heart and respiratory rates per subject and session were measured using physiological signals. This measurement was used to define a heart rate band (mean frequency +/−0.05 Hz) and a respiratory rate band (mean frequency +/−0.1 Hz) within which the frequency analysis measurements were made. Frequency analysis of the MRI signals was then performed using the multitaper approach (spectral resolution = 0.2 Hz, window length = 30 s, step = 1 s, time half-bandwidth product = 3, number of tapers = 5)^13^. For voxel-wise frequency analysis, multitaper spectrogram was calculated on the detrended MRI signal from each voxel. Maximal power values were extracted in both cardiac and respiratory frequency bands for each acquisition. Brain maps were constructed from log-transforms of these power values, normalized subjectwise by the maximal value of a subject. The voxel-wise maps for the 16 acquisitions, for which the T1w acquisition was done, were overlaid on the anatomical T1w acquisition to localize the regions that are most sensible to cardiac or to respiratory oscillations (Fig. 2). Only the values greater than 80% of the maximum power are shown. The color intensities match the amplitude of the MRI signal at the corresponding frequency.

For the ROI analysis, multitaper spectrogram was calculated on the detrended mean ROI signal. Maximal values of the spectral peaks were extracted as in the voxel-wise approach. These values were normalized by dividing the maximal value of the spectral peak at the cardiac or respiratory frequency by the integral of the spectrum of the signal between 0 and 2 Hz. These normalized peak amplitudes were then compared with paired permutation t-tests with 5000 repetitions, using permuco package in R (Fig. 3).

## Results

###  Physiological cycle reflected in the fMRI signal

The synchronized grand mean and the random grand mean are plotted in Fig. 1B. The PPG synchronized grand mean shows a significantly greater modulation than the random grand mean. The fMRI GS is in phase with the PPG signal and shows significant modulation at its peaks. The signal obtained in the MCA region shows important variability and is not found significantly different from the random grand mean. The signal from VR region is significantly modulated and inversed in phase with respect to the PPG signal. The signal from the CSF region shows a significant modulation and presents two oscillations per one PPG cycle.

The respiratory-induced variations on fMRI signal are more pronounced than the cardiac noise. The respiratory cycle synchronized grand mean shows a significantly greater modulation than the random grand mean. The respiratory modulation of GS is significant and peaks during the inspiration phase, the same modulation is observed in the MCA region. The signal from the VR region does not show a significant statistical difference from the random grand mean due to large variability, though it fluctuates visibly at the respiratory frequency. The synchronized mean signal from the CSF region shows a subtle modulation of the signal that is statistically different from the random mean. The amplitudes of the fMRI signal modulation in the MCA and GS by respiration are greater than those induced by the cardiac pulse.

### Spatial distribution of the physiological effects

In the frequency domain, Fig. 2 shows that the voxels mostly affected by cardiac or by respiratory cycles are not localized in the same regions. Cardiac noise is most pronounced in the CSF and VR regions. Respiratory noise is greater in regions close to MCA, as well as the ventricles and VR regions, and in the periphery of the brain, especially in the frontal part.

The permutation t-test shows a significant difference between the amplitudes of the spectral peaks at the cardiac and respiratory frequencies for the GS (t = 5.967; *p* < 0.001; see Fig. 3) and MCA area (t = 3.361; *p* = 0.001), but not venous return (t = −0.332; *p* = 0.749) or ventricles (t = −0.377; *p* = 0.7114).

## Discussion

This study evaluated the respective impact of the cardiac and respiratory cycle on the fMRI signal in human brain. The fMRI signal presents a modulation aligned with both cardiac and respiratory cycles. Cardiac pulse is reflected differently in different ROIs. The main peak of the GS fluctuates in phase with the cardiac cycle, while venous return is opposite in phase. The ventricular CSF oscillation is biphasic at each cardiac cycle. The respiratory cycle induces oscillations in the fMRI signal in the brain ROIs that are greater in amplitude. The GS and MCA regions are significantly more affected by the respiratory cycle.

The physiological cycles can modify the fMRI signal through multiple mechanisms. First, the BOLD effect, which is sensitive to the concentration of dioxygen in blood. Then, the changes in tissue composition in a given voxel, for example due to dilation or constriction of blood vessels can modify the intensity of signal in a given voxel. The cranial volume is limited and shared between the brain tissue, blood and CSF. According to the Monro-Kelly hypothesis^14^, as the fluids are incompressible, the blood inflow and outflow created by the cardiac pulse or breathing cycle constantly modify volume redistribution between tissues in the cranium. This volume redistribution between tissues is associated with the motion of the brain tissue in the cranial volume^15,16^, so this motion can also be the source of change in the fMRI signal. Finally, as we regard only one slice of the brain during this study, we consider that the observed change in the fMRI signal can also be due to the inflow effect. The inflow effect emerges as a result of the movement of fluid into or out of a slice, transporting different magnetization states from one location to another^17^. The increase in signal arrives when the bloodstream that has not yet been exposed to the radiofrequency pulse enters from beyond the imaging volume^18^. The inflow effect in this study evidences for the flow of liquids in the cranium and might be attributed uniquely to the use of one-slice acquisition, however, the inflow effect is also present in the whole-brain recordings even in the center of the volume^19^. The overall amplitude of the signal in different ROI depends on the tissue composition of the ROI, that have different magnetic properties.

### Cardiac cycle effect on fMRI signal

**Fig. 4:**
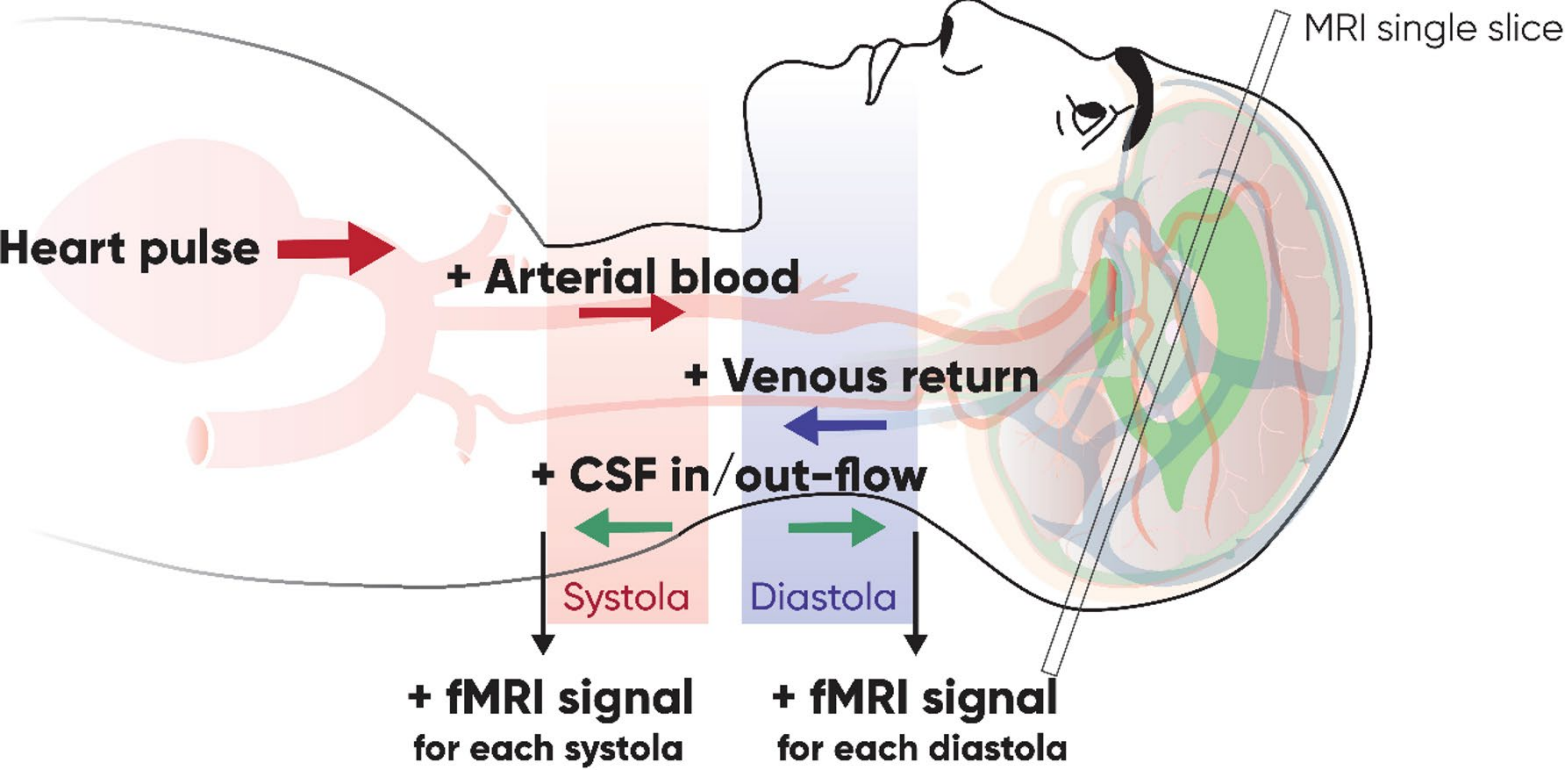
Illustration of physiological processes during each cardiac systole and diastole that modify the fMRI signal.

The differential effect of cardiac cycle on the fMRI signal from different brain regions reflects the undergoing physiological processes (Fig. 4). Every systole pushes the arterial blood to the brain arteries. Cerebral arteries and capillaries expand for the inflow of oxygenated blood^20^. This arrival of fresh oxygenated blood increases the fMRI signal mostly through the inflow effect^17^. It is interesting to notice that the main peak of the GS region signal is slightly in advance according to the PPG signal. This can be explained by the placement of the PPG on the finger: the path for blood to make from the heart to the head is shorter than the way from the heart to the finger, and so the PPG signal is in slight phase retard. Every diastole facilitates the outflow of the venous blood from the brain, so this deoxygenated blood flow creates the increase in the fMRI signal due to the inflow effect^17^.

The signal from the venous region has roughly a half-cycle phase difference with the PPG signal. This shows that the fMRI signal can be affected by both the systolic and diastolic phases, depending on the region. This observation contrasts with the assumptions that the cerebral effects of the cardiac cycle are mainly driven by the systolic phase, as proposed by Kassinopulous and Mitsis in 2021^21^. Notably, the authors^21^ report a similar phase shift of approximately half a cardiac cycle between the Card-RETROICOR model and Cardiac Pulsatility Model when adjusting their models via the phase offset. Combined, these observations support the view that one model may be more sensitive to physiological noise from venous outflow and the other from arterial inflow.

The amplitude of the signal from the venous area at the cardiac frequency is greater than the amplitude of the signal in the area containing arteries, because it is mostly the deoxygenated blood that affects the BOLD effect^18^, moreover, veins have a greater compliance than arteries, that leads to greater modifications of blood volume and thus, of the fMRI signal. The CSF ventricular flow is coupled with the cerebrovascular hemodynamic oscillations^8,22,23^. Interestingly, the CSF signal presents two oscillations with each cardiac pulse. The peaks of the CSF oscillation are aligned both with the peaks of the VR region and the peak of the PPG signal, respectively. This is again due to the inflow effect that increases the fMRI signal twice per cardiac cycle: the arrival of the arterial blood at the systole provokes the outflow of the CSF from the cranium and the venous blood leaving the cranium during the systole leaves place for the inflow of the CSF to the ventricles. This double-peak fluctuation of the CSF coupled to the cerebrovascular oscillations was also appreciated in a recent study by Yang and collaborators^8^. The GS roughly represents the sum of signals from three other ROI, being in phase with the cardiac pulse it keeps an attenuated double-peak pattern.

###  Respiratory cycle effect on fMRI signal

**Fig. 5:**
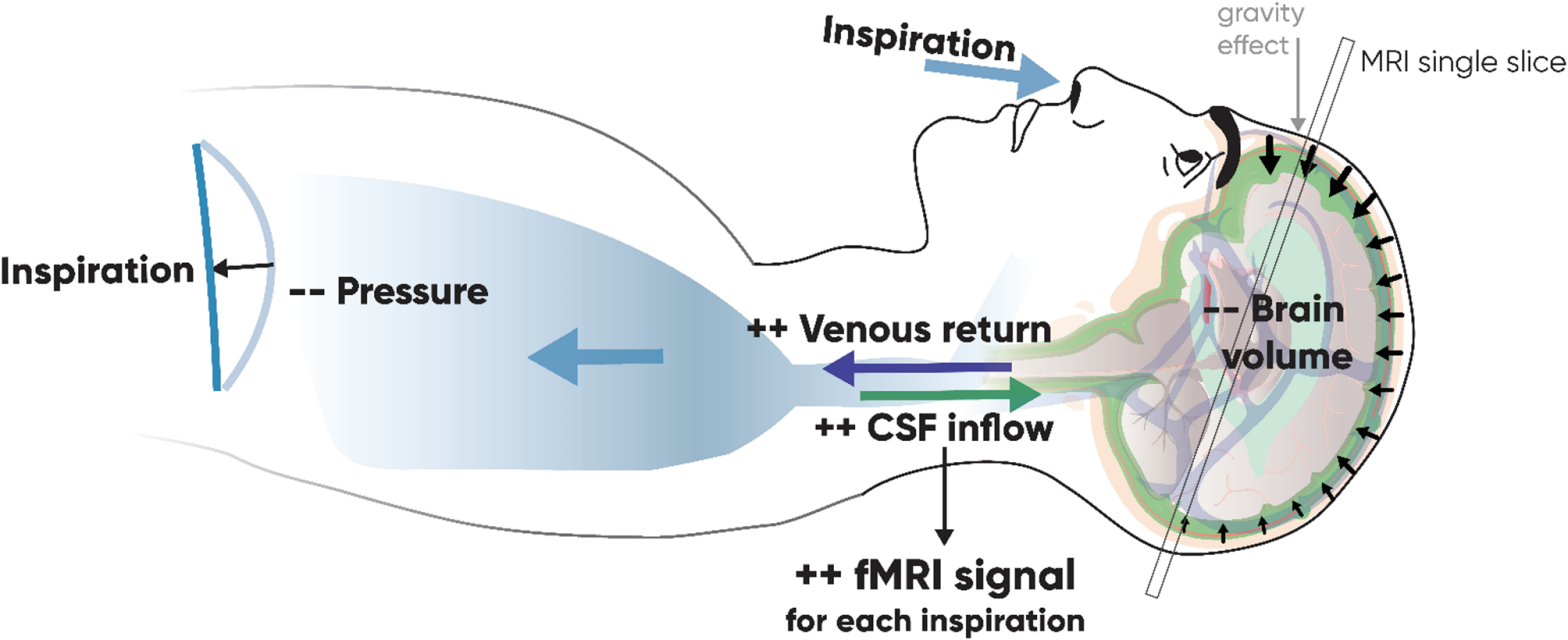
Illustration of physiological processes during each inspiration that modify the fMRI signal.

The breathing cycle induces significant modulation of the fMRI signal in the GS and the MCA regions. The respiratory effect is more homogenous in phase between the ROI than the cardiac effect. The fMRI signal from the MCA region peaks during the inspiration and is minimal at the end of the expiration. As the GS, the MCA region, is composed of different substances: arteries with oxygenated blood, grey matter and the extracerebral CSF, so we suggest the effect on this region is mostly due to the CSF flow in the subarachnoid space. The inspiration is produced by the contraction of the diaphragm, which decreases the pressure in the thorax (Fig. 5). This facilitates the outflow of the venous blood from the brain^24^. This venous emptying leads the brain to slightly shrink in volume, so the CSF inflows to the subarachnoid space at the contours of the brain. Indeed, inspiration has been shown to be accompanied by the upward flow of CSF on the spinal canal^25^ coupled with the venous outflow^26–28^. The expiration liberates this negative thorax pressure, which allows the cerebral veins to refill with venous blood, pushing the CSF out of the cranial volume.

Figure 2 shows that the respiratory effect is mostly localized in the veins and ventricles, and at the contours of the gray matter and following the sulcus, especially in the frontal lobe. This last localization of the effect supports our suggestion that the respiratory effect on the fMRI signal is partly mediated by the inflow of CSF at the contours of the gray matter. We have shown that the brain diminishes in volume during the inspiration, and as it floats in the CSF, in subjects lying supine, gravity pulls the brain to the occipital part of the head. Therefore, more CSF can be found at the frontal part of the cranium, which strongly increases the signal in this area.

The GS contained a significantly greater respiratory effect, than the cardiac effect. This finding is supported by a recent study using the MREG that found the respiratory effect in the BOLD signal to be greater than the cardiac one^11^. We suggest that respiration affects the fMRI signal not by the head movement as commonly assumed^4,7^, but through the change in flow of liquids.

### Limitations

The major limitation for the characterization of the effect of breathing and cardiac activity in fMRI signal is the difficulty of measuring the physiological component in the signal composed of neuronal activation and the physiological components both of which are unknown. Commonly, the noise component is modeled. We used an averaging approach that permits revealing the part of the signal that is synchronized to a trigger while nulling out other non-synchronized components. This method is suitable and particularly robust for both time-locked and phase-locked cycles^29^. This might be a mild limitation to the study, if the cardiac and respiratory responses are not time- and phase-locked.

The averaging technique for the ROIs is most robust for regions with homogenous composition like the veins and ventricles. The method might be slightly less demonstrative for the regions with heterogenous tissue composition, like the MCA. The signal fluctuations due to the cardiac cycle detected at the MCA region are visible, but not statistically significant due to a larger variability caused by heterogenous composition of the region.

The respiratory depth has not been taken into account, which might have added variability to the averaged cycles, limiting our ability to reveal the breathing effect in the fMRI signal. However, this is a common limitation for most imaging studies, as a pneumotachograph is rarely used in a scanner, and using the respiratory belt on the chest or abdomen permits to only approximate the respiratory depth with its measurements depending on the placement, morphology of the participant and their respiratory pattern (e.g. abdominal and thoracic expansion might very between subjects).

Finally, this study shows physiological effects on the fMRI signal primarily mediated by the inflow effect^19,30^, given the one-slice approach used here. This aligns well with the aim of the present study, which is to characterize the physiological processes occurring within the skull during fMRI acquisition. However, this might not be directly translatable to the common fMRI protocols that use the 3D volumes. For the evaluation of the physiological effect in standard fMRI conditions, an additional study is required using 3D acquisitions of long duration that will allow for acquiring enough epochs required for the averaging technique.

## Conclusion

The findings of this study demonstrate that physiological noise is heterogeneous across the brain in amplitude and phase. The cardiac-induced oscillation has different, opposite in phase, effects on the fMRI signal in the regions close to arteries and the regions close to veins. Another finding of this study is that the respiratory impact on the fMRI signal is greater in amplitude than the cardiac impact. Moreover, the results highlight the origin of the respiratory effect on the fMRI signal oscillations, which is mediated rather by the flow of blood and CSF during the respiratory cycle, and not the movements of the head. Altogether, our results support the use of methods that account for voxel-wise phase information, such as RETROICOR, over techniques based on a single, global PRF applied uniformly across the brain.

## Data Availability

The data that support the findings of this study are openly available at ResearchGate at http://doi.org/10.13140/RG.2.2.35703.33444.
